# Supplementary material for: Chromothripsis during telomere crisis is independent of NHEJ, and consistent with a replicative origin
Source: Genome Res. 2019 May;29(5):737–49. doi: 10.1101/gr.240705.118 (PMC6499312; doi:10.1101/gr.240705.118)
Supplement: Supplemental Material [file supp_gr.240705.118_Supplemental_file_1.zip › contigs/annotated_contigs/DB81/contig.2.DB81_length_482_mean_cov_6.14315352697.docx]

**DB81_length_482_mean_cov_6.14315352697**

```
TTCTCTATACAACCGTGAGCAAGTTCTTTACTTCTCTGGGTTTCCAAGTCTATATCTGCACAATGAGAGATTTGGGCTACATAGTTTCT
 >chr13:61701708-61702017 - E=1e-166 p=3e-02
AAGGTCTTTTTTAGCCCTAAATTTTTGAAAGTCAAATAATATTCCATAGAAGTTACTTCAGTTATTACTCTTCCAATCACATACTTATA

AATGCCTTAATGGGCTGTGTGTGGAATCTTTAGTGTAGTAGCTTTTTGGTTAAAGATGAGGGTGGAATGAGATAGCTGCCGAGATCATT

ACAGTTTCATGTTACATTTTCCTTGCCCCATATATATATAT|ATA|GCTAAAATTAAAAATGCTGACCATACCAAGTACTGGGGAGGAT
                                          >chr13:61342911-61343085 - E=4e-93
GTAGAGCAACTTGAACTCCCACACACTGTTGGTTGAAATGTGAAGTGGCAGCACTATTCACAATAGCAAAGACATGGTCAGCATAAATG

CCCATCAATGAGAGACTGGGATAAAGAAAATGTGATATA
```
